# Consequences of COVID-19 pandemic lockdown on emergency and stroke care in a German tertiary stroke center

**DOI:** 10.1186/s42466-021-00118-z

**Published:** 2021-03-31

**Authors:** Robin Jansen, John-Ih Lee, Bernd Turowski, Marius Kaschner, Julian Caspers, Michael Bernhard, Hans-Peter Hartung, Sebastian Jander, Tobias Ruck, Sven G. Meuth, Michael Gliem

**Affiliations:** 1 Department of Neurology, Medical Faculty, Heinrich-Heine-University, Moorenstr. 5, 40225 Düsseldorf, Germany; 2 Department of Diagnostic and Interventional Radiology, Medical Faculty, Heinrich-Heine-University, Düsseldorf, Germany; 3 Emergency Department, University Hospital, Heinrich-Heine-University, Düsseldorf, Germany; 4 Brain and Mind Centre, University of Sydney, Sydney, Australia; 5 Department of Neurology, Medical University of Vienna, Vienna, Austria

**Keywords:** Stroke, COVID-19, Lockdown, Pandemic

## Abstract

**Background:**

COVID-19 pandemic caused a decline in stroke care in several countries. The objective was to describe lockdown stroke care in a tertiary stroke center in Düsseldorf, Germany near Heinsberg, a German hot spot for COVID-19 in spring 2020.

**Methods:**

In a retrospective, observational, single-center study, we compared all patients treated in our emergency department (ED), patients seen by a neurologist in the ED, ED patients suffering from ischemic and hemorrhagic strokes and transient ischemic attacks (TIAs) as well as stroke patients admitted to our stroke unit during lockdown in spring 2020 (16 March 2020–12 April 2020) to those cared for during the same period in 2019 and lockdown light in fall 2020 (2 November – 29 November 2020).

**Results:**

In spring 2020 lockdown the mean number of patients admitted to our ED dropped by 37.4%, seen by a neurologist by 35.6%, ED stroke patients by 19.2% and number of patients admitted to our stroke unit by 10% compared to the same period in 2019. In fall lockdown light 2020 effects were comparable but less pronounced. Thrombolysis rate was stable during spring and fall lockdown, however, endovascular treatment (EVT) rate declined by 58% in spring lockdown and by 51% in fall lockdown compared to the period in 2019.

**Conclusions:**

Our study indicates a profound reduction of overall ED patients, neurological ED patients and EVT during COVID-19 pandemic caused lockdowns. Planning for pandemic scenarios should include access to effective emergency therapies.

## Background

Ischemic stroke is a leading cause of persistent disability in Western societies [1]. Established therapies comprising stroke unit treatment [2], i.v. thrombolysis (IVT) with rt-PA (recombinant tissue plasminogen activator) [3] and endovascular therapy (EVT) [4] can increase the likelihood of a favourable outcome when patients present immediately after symptom onset.

With the worldwide spread of Severe-Acute-Respiratory-Syndrome-Corona-Virus-2 (SARS-Cov-2), several reports were published on a significant decrease in the number of patients treated with acute medical conditions such as stroke or myocardial infarction [5–8]. To date the exact reasons remain unclear but reduced emergency department (ED) visits due to nation-wide lockdown rules with social distancing, patients’ fear of getting infected with SARS-CoV-2, and changes in the perception of hospitals during pandemic are possible explanations [7–9]. While restrictions are still in place and lockdown rules upheld, the effects are incompletely understood yet. With the nationwide first two deaths reported on 9 March 2020 in North Rhine-Westphalia, Germany, the State government ordered the closure of schools and kindergarten as early as the 16 March 2020, and one day later the shutdown of stores not essential for daily living. First lift of restrictions was declared for 11 May 2020. After a brief decline in the incidence of SARS-CoV-2 in summer, the federal government decided to shut down again when numbers rose in fall, in a “lockdown light” scenario from 2 November until 15 December 2020 with less severe rules compared to the spring lockdown.

In this retrospective, observational single-center study we provide a brief status report from the University Hospital Düsseldorf. Düsseldorf is the capital of Germany’s most densely populated state North-Rhine-Westphalia (17.93 million residents) with 612.000 inhabitants and three stroke units. The University Hospital Düsseldorf is the only tertiary stroke center in Düsseldorf providing EVT for an area of 1.000.000 inhabitants with a comprehensive stroke unit consisting of 24 beds including 12 monitoring and 12 non-monitoring beds. It takes care of approximately 1.000 patients per year in non-COVID-19 periods.

Düsseldorf is located 58 km apart from the district of Heinsberg. This region was the first to be classified as a particularly affected area in Germany in spring 2020 by the Robert Koch Institute, the national center for infectious diseases. We report on the number of patients admitted to our interdisciplinary ED and provide a detailed analysis of stroke patients during the coronavirus disease 2019 (COVID-19) pandemic lockdown-periods. Our interdisciplinary ED primarily treats all emergencies except for cases of ophthalmology and gynecology and obstetrics. These specialties have their own emergency premises in other buildings.

## Methods

**Fig. 1:**
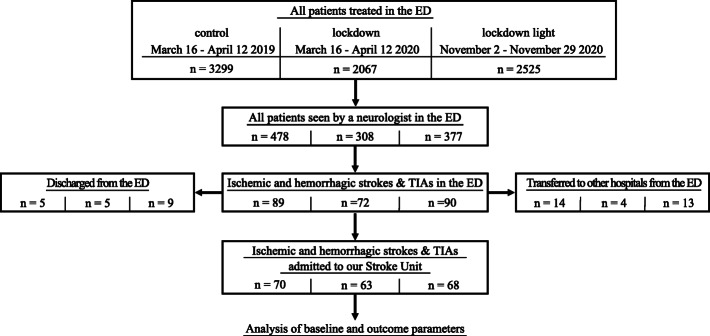
Flowchart of analyzed patients during COVID-19 spring lockdown (16 March – 12 April 2020) comparative time period one year ago (16 March – 12 April 2019) and lockdown light (2 November 2020–29 November 2020)

We retrospectively compared all patients treated in our ED, patients seen by a neurologist in the ED, patients suffering from ischemic and hemorrhagic strokes and transient ischemic attacks (TIAs) seen in the ED as well as stroke patients admitted to our stroke unit during spring lockdown (16 March 2020–12 April 2020) to patients treated within fall lockdown light (2 November – 29 November 2020) and during 16 March – 12 April 2019 (Fig. 1). Furthermore, in order to extend the data for fall lockdown we compared data to the period from 30 November – 27 December 2020. Every ED and ED stroke patient seen by a neurologist was marked by the neurologist in the electronic patient files, which have been implemented since 2014 for ED patients and since 2019 for stroke patients. The electronic patient files retrieved all ED patients seen by a neurologist and stroke patients of the ED for the different periods. The assessing method did not differ from 2019 and 2020. Routine medical care data were collected for quality control measures and were analyzed retrospectively in an anonymized (number of patients including strokes and TIAs treated in the ED) and pseudonymized (data of patients admitted to our stroke unit) manner. Data collection was approved by the local Ethics Committee of the Medical Faculty of the Heinrich Heine University Düsseldorf (#4042). For the observational retrospective analysis of anonymized and pseudonymized routine care data a separate written informed consent could be waived according to the local Ethics Committee.

### Outcome assessment

The number of patients per day was quantified for each group.

Clinical outcome of patients admitted to the stroke unit was assessed using modified Ranking Scale (mRS) and National Institutes of Health Stroke Scale (NIHSS) at discharge. Mortality during the hospital stay of patients admitted to the stroke unit was determined.

### Statistical analysis

SPSS Statistics 20 (IBM, Armonk, NY) and GraphPad Prism™ software (GraphPad 5 Software Inc., La Jolla, CA) were used for statistical analysis. Continuous data were analyzed by analysis of variance with Bonferroni correction (normally distributed) or Kruskal-Wallis-test and post-hoc Mann Whitney U test with Bonferroni correction (non-normally distributed). Between-group comparisons for categorical data were analyzed using Kruskal-Wallis-test and post-hoc Mann Whitney U test with Bonferroni correction. Daily admissions were tested by one-way ANOVA with Bonferroni correction, other tests were two tailed and results were assumed statistically significant at *p* < 0.05. Non-significant differences were indicated by ns.

## Results

### ED patients

**Fig. 2:**
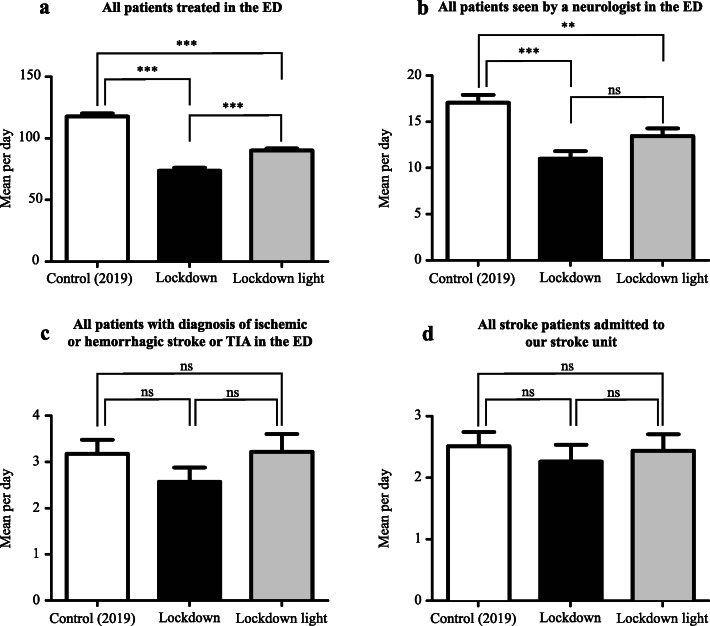
Comparison of mean patient numbers per day in control vs lockdown and lockdown light period (**a**) treated in the ED (mean 117.8 vs 73.8, *n* = 3299 vs *n* = 2067 reduction 37.4% and vs lockdown light with mean 90.18 and *n* = 2525, reduction 23.45%, *p* < 0.0001), (**b**) seen by a neurologist in the ED (mean 17.1 vs 11, *n* = 478 vs *n* = 308, reduction 35.6%, p < 0.0001 and vs lockdown light with mean 13.46 and *n* = 377, reduction 21.2%, *p* < 0.01), (**c**) with diagnosis of ischemic or hemorrhagic stroke or TIA in the ED (mean 3.2 vs 2.6, *n* = 89 vs *n* = 72, reduction 19.1%, and vs lockdown light with mean 3.2 and *n* = 90, non-significant increase by 1.1%) (**d**) with diagnosis of ischemic or hemorrhagic stroke or TIA admitted to our stroke unit (2.5 vs 2.3, reduction 10% and vs lockdown light with mean 2.4 by 2.9%. Daily admissions were tested by one-way ANOVA with Bonferroni correction. Results were assumed statistically significant with * = *p* < 0.05. ** = *p* < 0.01.*** = *p* < 0.0001. Ns indicates non-statistical significance

Compared to the same period in 2019, the mean number of ED visits significantly dropped in spring lockdown from 117.8 per day [95% CI: 112.8–122.9] by 37.4% to 73.8 per day [95% CI: 68.7–79.0] and to 90.18 by 23.45% [95% CI: 86.8–93.5] during fall lockdown light in 2020. The mean number of ED patients seen by a neurologist significantly dropped from 17.1 per day [95% CI: 15.4–18.8] by 35.6% to 11.0 per day [95% CI: 9.3–12.7] and still significant but less to 13.5 [95% CI: 11.8–15.2] by 21.2% in fall lockdown light as well [*p* < 0.01] (Fig. 2a, b).

### Stroke patients

From 16 March to 12 April 2019, the mean number of ED stroke patients was 3.2 per day [95% CI: 2.6–3.8]. During lockdown in spring, the mean number of ED stroke patients dropped by 19.1% to 2.6 patients [95% CI: 1.9–3.2], whereas in fall lockdown light patient numbers with a mean of  3.2 per day [95% CI: 2.4–4.0] were similar to 2019.

The mean number of patients admitted daily to our stroke unit was 2.5 [95% CI: 2.0–3.0] in 2019 and dropped by 10% to 2.3 patients [95% CI: 1.7–2.8] in spring lockdown and in fall lockdown light by 2.9% to 2.4 [95% CI: 1.9–3.0] respectively, which both did not reach statistical significance (Fig. 2c, d).

Extending the lockdown light analysis to December 2020 revealed comparable results for ED patients and stroke patients (data not shown). Stroke or TIA patients not admitted to our stroke unit were either transferred to other hospitals mostly because of limited capacity or discharged from the ED (Fig. 1). Approximately 50% of these discharged patients at each time point left the ED against medical advice (2019: 3 out of 5, spring lockdown: 2 out of 5, fall lockdown: 5 out of 8). The other reason was primarily the completion of the diagnostic workup in an outpatient setting.

Concerning the patients treated in our stroke unit, baseline characteristics did not differ between patients treated in 2019 and 2020, except for a significantly elevated rate (+ 79.6%) and absolute number (+ 80.9%) of macroangiopathic/large artery arteriosclerosis stroke etiology in fall lockdown light compared to spring lockdown (Table 1). In addition, large vessel occlusions in spring lockdown showed a reduced rate (− 53%) and absolute number (− 56%) compared to 2019, but did not reach statistical significance (Table 1). Interestingly, while the rate of IVT was comparable (2019: 17 of 70 (24.3%), spring 2020: 15 of 63 (23.8%) and fall 2020: 14 of 68 (20.06%), *p* > 0.05), the relative rate of EVT (2019: 21 of 70 (30%), spring 2020: 8 of 63 (12.7%) and fall 2020: 10 of 68 (14.7%)) was significantly reduced by 58% from 2019 to spring 2020 and non- significantly by 51% from 2019 to fall 2020. A comparison to the 2019 monthly mean of IVT (14.5, 95% CI 8.9–20.1) and EVT (16, 95% CI: 8.8–23.5) yielded comparable results (spring 2020: IVT + 17.2%, EVT − 50%, fall 2020: IVT -3,4%, EVT – 37.5%). 9 out of 21 EVT patients in 2019, 5 out of 8 EVT patients in spring 2020 and 2 out of 10 EVT patients in fall 2020 were admitted to us from other hospitals. Interestingly, the decline in overall EVT correlated with a reduced number of directly transferred EVT patients and reduced numbers of patients with large vessel occlusion (Table 1).

**Table 1:** Baseline and outcome parameters of patients admitted to the stroke unit

Time interval	A March 16–April 12 in 2019, same time interval one year before COVID-19 spring lockdown		B March 16–April 12 in 2020, COVID-19 spring lockdown		C November 2–November 29 in 2020, COVID-19 fall lockdown light		*p*-value, test
n=	70		63		68		
Median age (IQR)	79 (69–85)		77 (64–82)		80 (62–85)		ns, ANOVA
Male gender	32 (45.7%)		30 (47.6%)		32 (47.1%)		ns, Kruskal-Wallis-Test
Vascular risk factors							
Arterial hypertension	55 (78.6%)		44 (69.8%)		49 (72.1%)		ns, Kruskal-Wallis-Test
Diabetes Mellitus	14 (20%)		18 (28.6%)		17 (25.0%)		ns, Kruskal-Wallis-Test
Hyperlipidemia	27 (38.6%)		18 (28.6%)		23 (33.8%)		ns, Kruskal-Wallis-Test
Coronary heart disease	14 (20%)		9 (14.3%)		13 (19.1%)		ns, Kruskal-Wallis-Test
Smoking	5 (7.1%)		10 (15.9%)		8 (11.9%)		ns, Kruskal-Wallis-Test
Peripheral artery disease	3 (4.3%)		5 (7.9%)		3 (4.4%)		ns, Kruskal-Wallis-Test
No vascular risk factors	6 (8.6%)		4 (6.3%)		0 (0%)		ns, Kruskal-Wallis-Test
Type of stroke							
Cerebral infarction	53 (75.7%)		47 (74.6%)		53 (77.9%)		ns, Kruskal-Wallis-Test
TIA	11 (15.7%)		10 (15.9%)		11 (16.2%)		ns, Kruskal-Wallis-Test
Intracerebral hemorrhage	6 (8.6%)		6 (9.5%)		4 (5.9%)		ns, Kruskal-Wallis-Test
Ischemic stroke etiology							
Cardioembolic	23 (32.9%)		21 (33.3%)		13 (19.1%)		ns, Kruskal-Wallis-Test
Embolic stroke of undetermined source	6 (8.6%)		10 (15.9%)		19 (27.9%)		ns, Kruskal-Wallis-Test
Microangiopathic	12 (17.1%)		13 (20.6%)		6 (8.8%)		ns, Kruskal-Wallis-Test
Macroangiopathic/Large artery arteriosclerosis	10 (14.3%)		4 (6.3%)*		21 (30.9%)*		*p < 0.05 for B and C, Kruskal-Wallis-Test
Patent foramen ovale associated	5 (7.1%)		3 (4.8%)		3 (4.4%)		ns, Kruskal-Wallis-Test
Unknown ischemic stroke etiology	8 (11.4%)		6 (9.5%)		12 (17.6%)		ns, Kruskal-Wallis-Test
Large vessel occlusion (ICA, carotid T, M1, M2, basilar artery)	25 (36%)		11 (17%)		15 (22%)		ns, Kruskal-Wallis-Test
Clinical and intrahospital management characteristics							
I.v. thrombolysis	17 (24.3%)		15 (23.8%)		14 (20.6%)		ns, Kruskal-Wallis-Test
Endovascular therapy	21 (30%)*		8 (12.7%)*		10 (14.7%)		*p < 0.05 for A and B, Kruskal-Wallis-Test
Admission within 4.5 h after stroke onset	21 (30%)		21 (33.3%)		32 (47.1%)		ns, Kruskal-Wallis-Test
Median onset to door time in minutes (IQR)	99.0 (57.0–445.25)	*n* = 32	120.5 (55.5–300.5)	*n* = 32	120 (59–684)	*n* = 37	ns, Kruskal-Wallis-Test
Median door to needle time in minutes (IQR)	47 (37.3–57.3)	*n* = 7	42 (20–62.5)	*n* = 13	40 (28.25–78.75)	*n* = 12	ns, ANOVA
Median door to groin puncture time in minutes (IQR)	77 (32–116)	*n* = 19	85 (36–85)	*n* = 7	121.50 (26.25–176.25)	*n* = 8	ns, ANOVA
Median NIHSS at admission (IQR)	4 (1–11)	*n* = 68	4 (1.8–10)	*n* = 62	5 (1.5–8)	*n* = 65	ns, Kruskal-Wallis-Test
Median NIHSS at discharge (IQR)	1 (0–4)	*n* = 59	1 (0–5)	*n* = 54	1 (0–5.5)	*n* = 61	ns, Kruskal-Wallis-Test
Median mRS at admission (IQR)	3 (1–5)	*n* = 70	3 (2–4)	*n* = 63	3 (2–4)	*n* = 68	ns, Kruskal-Wallis-Test
Median mRS at discharge (IQR)	2 (0–4)	*n* = 69	2 (1–4)	*n* = 63	2 (1–4)	*n* = 68	ns, Kruskal-Wallis-Test
Median In-hospital days (IQR)	6 (4–9)		6 (4–9)		7.5 (5–11)		ns, Kruskal-Wallis-Test
In-hospital deaths	9 (12.9%)		8 (12.7%)		7 (10.3%)		ns, Kruskal-Wallis-Test

Continuous variables are reported as medians with interquartile range (IQR), categorial variables are reported as absolute numbers and as proportion. Between-group comparisons for categorical data were analyzed using Kruskal-Wallis-test and post-hoc Mann Whitney U test with Bonferroni correction. Group comparisons for continuous data were performed with ANOVA and post-hoc Bonferroni correction (normally distributed) or Kruskal-Wallis-test and post-hoc Mann Whitney U test with Bonferroni correction (non-normally distributed). All tests were two tailed and results were assumed statistically significant with p < 0.05. Ns indicates non-statistical significance

Outcome parameters and in-hospital deaths of patients admitted to our stroke unit did not differ between the groups. None of the stroke patients in these periods suffered from COVID-19. Only two patients were initially treated on a separate ward due to COVID-19 suspicion on admission in spring 2020. None of those had an indication for IVT or EVT.

Between 16 March and 12 April 2020 the mean number of COVID-19 patients treated in our hospital per day was 20 with a mean of 8 patients per day on our intensive care unit. During 2 November and 29 November 2020 the mean number of COVID-19 patients treated in our hospital per day was 53 with a mean of 14 patients per day on our intensive care unit.

## Discussion

Recent literature suggests that during the COVID-19 pandemic, numbers of stroke patients admitted to hospitals in different regions of the world decline [7–14]. Our single-center retrospective observational study during COVID-19 pandemic lockdown additionally to stroke patients comprises all ED visits and all ED patients seen by a neurologist. It documents an overall reduced utilization of the ED. It could be shown that total daily ED visits and ED patients seen by a neurologist were more severely reduced than stroke admissions. During the COVID-19 lockdown the University Hospital Düsseldorf as a tertiary center was reorganized for COVID-19 patients with special COVID-19 intensive care unit wards, non-intensive care COVID-19 wards and suspicion of COVID-19 wards. However, stroke care of patients without COVID-19 suspicion was unaffected and not limited. Therefore, we could not recognize structural limitations in our hospital that would explain our observations.

Reasons for a drop in ED visits and stroke admissions might be a consequence of social distancing as suggested by Hoyer et al. in their multicenter study and by Richter et al. in their large nationwide German stroke patient care analysis [7, 8] or the reduced public transportation and patients’ fear of getting infected with SARS-CoV-2 in the hospital as Zhao et al. [9] assume in their study. The latter is well in line with reports from the United Kingdom showing that, in contrast to a perceived reduction in the number of hospital admissions, ambulance callouts for stroke and myocardial infarction did not decrease [15]. The smaller decline in stroke admissions as compared to overall ED visits might be due to the severity of the clinical condition. Interestingly, the reduction seen in our sample was much less pronounced than reductions observed during the first COVID-19 spring lockdowns in Piacenza, Italy (88% reduction of stroke patients presenting to the ED) [11], in Aragón, Spain (71% reduction of stroke patients) [10], in a Chinese registry (38% reduction of stroke admissions) [9], and even smaller than in New Jersey, US with a reduction of daily stroke admissions from 1.82 to 1.13 (38%) [12]. In contrast, in a further German single-center study the absolute daily number of Code Stroke referrals even remained stable [16].

These differences might be explained by a less stressed health system and less severe lockdown restrictions in Germany and might fuel discussions about health system reserve capacities and side effects of different lockdown intensities.

Furthermore, during fall “lockdown light” we observed significantly more large artery arteriosclerosis induced ischemic strokes compared to the spring lockdown, which might be primarily due to the small sample size and individual stochastic fluctuation.

In the fall lockdown scenario including also numbers of December 2020 more patients overall and seen by the neurologist presented to the ED compared to spring lockdown, but still significantly less than in 2019. Reasons for higher ED presentations in the fall “lockdown light” might include adaptation processes to the pandemic situation with diminished anxiety and less strict lockdown rules during fall “lockdown light”.

On the other hand, while our IVT rate was stable (23.8% vs 24.3% vs 20.6%), which was in contrast to other observations [13, 17–19], but in line with the nationwide German cohort study [8], the rate of large vessel occlusion dropped not significantly along with a significant reduction of our EVT rate and absolute number between 2019 and the spring lockdown of EVT by 58 and 62%, respectively. Our observed reduction of EVT is beyond the 21% decline reported from a registry in France [20] or the 23% decline reported from China [9]. Stable daily IVT numbers were also reported in another German study [16], which in contrast to our data also reported stable EVT numbers [16] indicating regional differences. Furthermore, the large nationwide cohort study with data from 1463 German hospitals found an even higher EVT rate during the spring lockdown compared to prepandemic control periods [8].

The proximity of our hospital to the district of Heinsberg, which was particularly affected with COVID-19 patients in spring 2020 and borders the area of our thrombectomy service, might be a reason for changed behavior of stroke patients and might have influenced transfer decisions from this area during the spring lockdown.

We cannot exclude individual stochastic fluctuations in patients’ admissions; however, also compared to 2019 the EVT reduction approached 50% in spring lockdown and 37.5% in fall lockdown. In all three periods almost equal numbers of potential EVT candidates did not go to the angiography suite (4 in 2019, 4 in spring lockdown and 3 in fall lockdown). Therefore, we can rule out that in the lockdown periods of 2020 the number of admitted EVT candidates not referred to the angiography suite was lower than in 2019. There may be different explanations for this decline of EVT. In preparation for a presumed influx of COVID-19 patients ED and intensive care capacities had been increased nationwide in part by deferring admission of patients for elective procedures and by enlarging intensive care unit space and facilities. Hence, EVT might have been performed also in hospitals that in non-COVID-19 times would have transferred their patients due to limited ED, interventional or intensive care capacities. Furthermore, the exchange and transfer of patients between hospitals was complicated due to COVID-19 restrictions, possibly preventing the performance of EVT in unclear cases.

On the other hand, we cannot exclude a protective effect of lockdown on stroke incidence by reducing some stress factors in everyday life in analogy to a doubled rate of myocardial infarctions during and normalized rate after watching a soccer game [21]. Further insight might be gained when analysis of stroke related death rates based on public health data is available.

A non-significant increase of the door to groin puncture time in fall lockdown compared to the other periods is mainly driven by the fact that a smaller number of patients was transferred from other hospitals for EVT (2019: 9 out of 21, spring lockdown: 5 out of 8, fall lockdown: 2 out of 10) in this period. Door to groin puncture time in our hospital is reduced in those patients due to stroke workup already done in the transferring hospital. Patients were then more rapidly transferred from our door to the angiography suite.

In addition, onset to door time in spring as well as in fall lockdown was not significantly extended compared to 2019, which may partly reflect the hesitation of patients to report as fast as possible to the ED in COVID-19 lockdown periods because of infection fear.

Our report has several limitations. We conducted a retrospective single-center study in a short time frame resulting in limited numbers of patients analyzed. However, as restrictions are ongoing and decisions on lockdown rules are currently discussed, we believe these data can offer useful information. In addition, we used data from our electronic patient files. Patients in the ED have been marked by the treating neurologist in the electronic patient files on a daily routine. Therefore, we do not expect a relevant amount of data missing. Furthermore, there are patients who had been discharged or transferred from the ED to other hospitals and could not be included in further analysis. In addition, long-term survival and long-term outcomes are not reported.

## Conclusions

Our study indicates a profound reduction of overall ED patients, neurological ED patients and EVT during COVID-19 pandemic caused lockdowns. Our data may contribute to informed decisions on how to prepare for future pandemic situations. We propose that the access to the emergency facilities of the medical system should be well planned, facilitated, and announced to the public in future pandemic scenarios to maintain highly effective emergency care as well as stroke care despite healthcare service reorganizations and to minimize deleterious side effects, poorer outcomes, and patients’ fear.

## Data Availability

The datasets used and/or analysed during the current study are available from the corresponding author on reasonable request.

## Abbreviations

COVID-19: Coronavirus disease 2019
ED: Emergency department
EVT: Endovascular treatment
IVT: Intravenous Thrombolysis
mRS: Modified Ranking Scale
NIHSS: National Institutes of Health Stroke Scale
rt-PA: Recombinant tissue plasminogen activator
SARS-Cov-2: Severe-Acute-Respiratory-Syndrome-Corona-Virus-2
TIA: Transient ischemic attack
